# Associations Between Social Capital and Depressive Symptoms Among College Students in 12 Countries: Results of a Cross-National Study

**DOI:** 10.3389/fpsyg.2020.00644

**Published:** 2020-04-29

**Authors:** Insa Backhaus, Andrea Ramirez Varela, Selina Khoo, Katja Siefken, Alyson Crozier, Edvaldo Begotaraj, Florian Fischer, Jascha Wiehn, Beth A. Lanning, Po-Hsiu Lin, Soong-nang Jang, Luciana Zaranza Monteiro, Ali Al-Shamli, Giuseppe La Torre, Ichiro Kawachi

**Affiliations:** ^1^Department of Public Health and Infectious Diseases, Sapienza University of Rome, Rome, Italy; ^2^Department of Social and Behavioral Sciences, Harvard T.H. Chan School of Public Health, Boston, MA, United States; ^3^School of Medicine, Universidad de los Andes, Bogotá, Colombia; ^4^Centre for Sport and Exercise Sciences, University of Malaya, Kuala Lumpur, Malaysia; ^5^Alliance for Research in Exercise, Nutrition and Physical Activity, University of South Australia, Adelaide, SA, Australia; ^6^Faculty of Health Science, MSH Medical School Hamburg, Hamburg, Germany; ^7^Department of Dynamic and Clinical Psychology, Sapienza University of Rome, Rome, Italy; ^8^School of Public Health, Bielefeld University, Bielefeld, Germany; ^9^Institute of Gerontological Health Services and Nursing Research, Ravensburg-Weingarten University of Applied Sciences, Weingarten, Germany; ^10^Institute of Public Health, Charité - Universitätsmedizin Berlin, Berlin, Germany; ^11^Department of Public Health, Baylor University, Waco, TX, United States; ^12^Graduate Institute of Sports, Leisure and Hospitality Management, National Taiwan Normal University, Taipei, Taiwan; ^13^Red Cross College of Nursing, Chung-Ang University, Seoul, South Korea; ^14^Departamento de Enfermagem, Centro Universitário do Distrito Federal (UDF), Brasília, Brazil; ^15^Departamento de Educação Física e Fisioterapia, Centro Universitário do Distrito Federal (UDF), Brasília, Brazil; ^16^Department of Physical Education, Sohar University, Sohar, Oman

**Keywords:** social determinants of health, social capital, mental health, depressive symptoms, university students, multilevel analysis

## Abstract

**Introduction:**

A mental health crisis has hit university campuses across the world. This study sought to determine the prevalence and social determinants of depressive symptoms among university students in twelve countries. Particular focus was placed on the association between social capital and depressive symptoms.

**Methods:**

A cross-sectional study was conducted among students at their first year at university in Europe, Asia, the Western Pacific, and Latin and North America. Data were obtained through a self-administered questionnaire, including questions on sociodemographic characteristics, depressive symptoms, and social capital. The simplified Beck’s Depression Inventory was used to measure the severity of depressive symptoms. Social capital was assessed using items drawn from the World Bank Integrated Questionnaire to Measure Social Capital. Multilevel analyses were conducted to determine the relationship between social capital and depressive symptoms, adjusting for individual covariates (e.g., perceived stress) and country-level characteristics (e.g., economic development).

**Results:**

Among 4228 students, 48% presented clinically relevant depressive symptoms. Lower levels of cognitive (OR: 1.82, 95% CI: 1.44–2.29) and behavioral social capital (OR: 1.51, 95% CI: 1.29–1.76) were significantly associated with depressive symptoms. The likelihood of having depressive symptoms was also significantly higher among those living in regions with lower levels of social capital.

**Conclusion:**

The study demonstrates that lower levels of individual and macro-level social capital contribute to clinically relevant depressive symptoms among university students. Increasing social capital may mitigate depressive symptoms in college students.

## Introduction

Over the past decade, a mental health crisis has challenged many university campuses across the world (Auerbach et al., 2018). A recent study showed that the occurrence of poor mental health among college students can be as high as 51% in some countries (Auerbach et al., 2018). Mental health issues during the college years have been associated with an increased risk of substance abuse, substantial impairment of quality of life and suicidal thoughts and behaviors (Pedrelli et al., 2016; Mortier et al., 2018). For many students, it also correlates with poor academic performance and dropping-out, which can deleteriously affect social mobility (Bruffaerts et al., 2018).

While several studies have documented trends in mental health issues among college students fewer have aimed to explain them, and there is still a knowledge gap regarding the underlying determinants of the increasing rates (Bruffaerts et al., 2018; Ngin et al., 2018). In light of the risks and consequences associated with mental health issues in college students, understanding what impacts their mental health is imperative. Traditional research exploring students’ mental health has focused primarily on individual-level characteristics such as age, gender and lifestyle behavior (e.g., heavy episodic drinking) as risk factors for depressiveness, but relatively little attention has been paid to wider social determinants of health (Geisner et al., 2012; Pedrelli et al., 2013; Ngin et al., 2018). More recently, social capital, broadly defined as features of social structures, including norms, interpersonal trust, and mutual support that act as resources for individual, has been identified as an upstream determinant of mental health (Fujiwara and Kawachi, 2008; Kawachi et al., 2008; Bassett and Moore, 2013). Cumulative evidence has shown that individuals with higher levels of social capital enjoy better mental health than individuals with lower levels of social capital. Yet, despite the mounting evidence regarding the importance of social capital on health the bulk of evidence to date has been conducted among adults and adolescents. The evidence about the association between social capital and health outcomes among college students remains limited (Morgan and Haglund, 2009; Borges et al., 2010).

Moreover, when determining factors associated with mental health, it is vital to take into account that individual determinants are incorporated in more distal contexts at the macro-level. Currently, most projections on student mental health as well as on social capital and health are limited to within-country studies rather than multilevel or cross-national studies (Steptoe et al., 2007). Depressiveness in college students, however, can vary significantly by geographic context. Therefore, cross-country research is needed to enable comparisons and identify possible levers from a broader perspective. The aim of this study was to investigate students’ mental health across a variety of countries and to examine the associations between depressive symptoms and social capital.

Therefore, we hypothesized that students reporting lower levels of social capital experience greater depressive symptoms, and that country-level differences in social capital can partly explain between-country variations in depressive symptoms.

## Materials and Methods

The present study reports findings from the Social Capital and Student Health Study (SPLASH study). The SPLASH study is an annual international survey examining mental health and related factors among undergraduate students across the world. The study received ethical approval by Institutional Review Boards and ethics committees at all participating institutions.

### Participants

The present study utilizes the 2018–2019 SPLASH study dataset, encompassed of self-reported data from about 4.200 university students across twelve countries in Europe (Albania, Germany, Italy, Kosovo, Switzerland), Asia (Malaysia, Oman, South Korea, Taiwan), the Western Pacific (Australia), and Latin and North America (Brazil, United States). At each institution that enrolled in the SPLASH study, a sample of degree-seeking students over the age of 18 years old were recruited.

### Procedures

In the present study, between one and two universities were sampled in each country and there were no exclusion criteria for institutional enrollment. All first-year students in the participating institution were invited to complete a self-administered questionnaire. The sampling scheme differed by country. Students from four universities (Brazil, Germany, Italy, and Oman) participated during a regular class lesson and students from the remaining universities participated in a web-based survey. Participation in the study was voluntary and anonymous. Before participating, students were informed that they could terminate participation at any point while filling out the questionnaire.

The sample size was calculated with a sensitivity of 95%, a margin of error of no more than ± 5% using the estimated prevalence of depressive symptoms (mild/moderate) in each country (i.e., each university) and the student enrollment at each university. For detailed information on the prevalence rates in each country, the number of students enrolled at each university and, the exact sample size calculation see the Supplementary Material.

### Measures

#### Sociodemographic Characteristics

Self-reported information was collected on a range of factors that have been identified as being relevant to an individual’s mental health, including age, gender, accommodation type during the semester, academic discipline and family socioeconomic status.

#### Depressive Symptoms and Suicidal Ideation

The outcome of interest was depressive symptoms. Depressive symptoms were measured using the Simplified Beck Depression Inventory (BDI-S) (Schmitt and Maes, 2000). The Beck Depression Inventory (BDI), in general, is one of the most widely used instruments for measuring depression and has excellent psychometric properties, including high internal consistency (α = 0.92) and demonstrated a lack of racial bias in university settings (Cassady et al., 2019). Measuring depressive symptoms in the university setting has been widely used and replicated by other authors (Cassady et al., 2019).

The BDI-S is a more efficient version of the BDI that has been shown to be no less reliable or valid (Sauer et al., 2013). It has 20 items and measures the severity of depressive symptoms on a six-point Likert response scale book-ended by 0 = ‘Never’ and 5 = ‘Almost Always’ (Schmitt and Maes, 2000). A single unweighted score for individual respondents can be computed by summing their responses for all items of the scale. The score can range from 0 (minimum score) to 100 (maximum score) (Schmitt and Maes, 2000).

Schmitt et al. (2006) have also provided standard values for detecting clinically relevant depressive symptoms, with a cut-off score at ≥35 representing clinically relevant depressive symptoms (Schmitt et al., 2006). The authors of the BDI-S have demonstrated high internal consistency (Cronbach’s α = 0.93). In the present study, Cronbach’s alpha was comparably high (α = 0.91).

The assessment of suicidal thoughts was based on item #9 of the BDI-S which asked students to indicate if they have had thoughts about killing themselves. The suicide item of the Beck’s depression inventory is considered a robust predictor of suicide attempts (Green et al., 2015).

#### Social Capital

Items drawn from the World Bank Integrated Questionnaire to Measure Social Capital (IQ-SC), a psychometric validated instrument, were used to measure social capital (Grootaert et al., 2004). Students were asked a wide range of questions relating to the “cognitive” and “behavioral” social capital dimensions. The cognitive dimension of social capital was assessed through five questions about the respondent’s: (a) trust in other people, (b) perceived helpfulness of others, and (c) perceptions of whether the one could borrow money from others in case of need. Four items were measured on a five-point Likert scale (e.g., 1 = ‘Agree’ strongly to 5 = ‘Disagree strongly’) and one question had a binary response option (e.g., 0 = ‘You can’t be too careful’ or 1 = ‘People can be trusted’). Composite scores for the individual five items were calculated by summarizing the individual sub item scores, such that a high score indicates higher levels of cognitive social capital. Individual scores for behavioral social capital, for example, can range from 0 to 18 and for cognitive social capital from 0 to 22.

The Cronbach’s alpha for the cognitive dimension was α = 0.71. The behavioral dimension of social capital was measured by the respondents (a) participation in community activities during the past twelve months, (b) time or monetary contribution to a community project, (c) belonging to a group, (d) having a close friend, and (e) getting together with people to have food or drinks in the past month. Items were either binary (yes/no) or Likert scale, with all scales recoded, where necessary, so that higher values represented higher levels of social capital. The Cronbach’s alpha for the behavioral dimension was α = 0.72.

#### Self-Rated Health

Self-rated health was assessed via the single item: “How would you rate your health in general?” and five-point scale response option (1 = ‘Excellent,’ 2 = ‘Very good,’ 3 = ‘Good,’ 4 = ‘Fair,’ 5 = ‘Poor’). For the analysis, the responses were dichotomized into fair/poor health versus the rest.

#### Perceived Stress

Perceived stress was measured using Cohen’s Perceived Stress Scale (PSS-10) (Cohen et al., 1983). It constitutes 10 questions, on a 5-point Likert response scale ranging from 0 = ‘Never’ to 4 = ‘Very often,’ on extent to which a respondent considers life stressful in the last month (Cohen et al., 1983). Individual scores on the PSS can range from 0 to 40 with higher scores indicating higher perceived stress. In particular, scores from 0 to 13, 14 to 26 and 27 to 40 represent the threshold for low stress, moderate stress and high perceived stress, respectively. The PSS-10 has been widely shown to demonstrate validity and reliability (Andreou et al., 2011). In the present study, the Cronbach’s alpha was acceptable with α = 0.76.

#### Alcohol Consumption and Heavy Episodic Drinking

Alcohol consumption and heavy episodic drinking was assessed using the Audit-C questionnaire (Bush et al., 1998). Scores for the AUDIT-C range from 0 to 12, with higher scores indicating a more hazardous drinking pattern (Bush et al., 1998). Heavy episodic drinking was defined as an AUDIT-C score of five or greater for men and four or greater for women. Several studies have found the AUDIT-C to be valid and reliable across various settings and different racial/ethnic groups (Seth et al., 2015). Cronbach’s alpha was high (α = 0.95). For the analysis, students were distinguished into low-risk and high-risk drinkers.

#### Physical Activity

Physical activity levels were measured using the short form of the International Physical Activity Questionnaire (IPAQ) (Craig et al., 2003; Hagströmer et al., 2006). The IPAQ has been recognized as a valid and reliable tool and consists of seven questions in which respondents are asked to report the number of days and the duration of their vigorous, moderate, and walking activity during the last week (Craig et al., 2003). A detailed description of the IPAQ scoring protocol is available elsewhere (Hagströmer et al., 2006).

### Country-Level Predictors

Since inequalities between different societies and nations are related to differences in economic development, countries were grouped according their level of purchasing power parity (PPP)-adjusted level of economic development as determined by the World Bank (2019). The World Bank classifies the world’s economies into four income groups: Low-income (not present in our study), lower-middle-income (Kosovo), upper-middle-income (Albania, Brazil, Malaysia), high-income (Australia, Germany, Oman, Taiwan, South Korea, Switzerland, United States). The World Bank determines the level of economic development by national income per person, or GNI per capita and by the classification threshold (World Bank, 2019).

### Data Analysis Strategy

We dichotomized the outcome according to BDI-S scores: not clinically depressed (<35) vs. clinically relevant depression ≥35). This reflects the use of the BDI-S in clinical practice as a screening tool to identify those who deserve further investigation (Nollett et al., 2019). Descriptive statistics were performed to characterize the sample and to determine the levels of social capital, self-rated health, depressive symptoms, perceived stress and health behaviors in each country.

A multilevel binary logistical regression was conducted to account for the hierarchical structure of the data (i.e., individuals nested within countries). Three models were performed. Model 1 included potential confounders of depressive symptoms (e.g., age, sex, and socioeconomic status) and Model 2 included the individual-level variables such as perceived stress and physical activity. In Model 3 country-level characteristics (e.g., economic development) were added. For all models, intra-class correlations (ICC) were calculated to measure the total variance in depressive symptoms that might be attributable to between-country variation (Level-2 units). The single equation can be written as follows:

logit(P)ij=γ+00γx10+ijγz01+jγx11zij+ju+0jux1jij

Here P_*ij*_ denotes the binary response variable that an individual will experience the outcome (i.e., clinically relevant depressive symptoms). The subscripts i and j reflect individual university students (at level 1) in the countries (at level 2). x_*ij*_ denotes the individual level predictors (e.g., social capital) and z_*j*_ indicates the country-level predictors (e.g., economic development). μ_0j_ represents the random residual for level 2. The segments γ_00_ + γ_10xij_ + γ_01zj_ + γ_11_x_*ij*_z_*j*_ and μ_0j_ + μ_1jxij_, represent the fixed effect and random part of the model, respectively.

Data analysis was performed using the statistical program STATA, version 15.0. Statistical significance level was set at *p* < 0.05.

### Sensitivity Analysis

To test the robustness of the results, two sensitivity analyses have been conducted to determine the influence of individual countries on the overall estimates. In the first sensitivity analysis countries with very high rates of depressive symptoms (e.g., Brazil) were excluded Supplementary Table S2), and the in the second sensitivity analysis a linear regression analysis using depressive symptoms as a continuous scale was performed (Supplementary Table S3).

### Findings

#### Sample Characteristics

Tables 1, 2 present the descriptive statistics for students with and without depressive symptoms. A total of 4228 first-year students participated in the survey. The total sample was comprised of students from Albania (*n* = 258), Australia (*n* = 397), Brazil (*n* = 549), Germany (*n* = 708), Italy (*n* = 402), Kosovo (*n* = 142), Malaysia (*n* = 444), Oman (*n* = 278), South Korea (*n* = 319), Switzerland (*n* = 251), Taiwan (*n* = 214), and the United States (*n* = 266). A more detailed Table including the mean age of students in each country can be found in the Supplementary Material (Supplementary Table S1).

**TABLE 1:** Sociodemographic characteristics of university students with and without depressive symptoms (*N* = 4228).

	Not clinically relevant (BDI-S < 35) %	Clinically relevant (BDI-S ≥ 35) %	*p*-value
**Total**	51.9	48.1	
**Gender**			
*Male*	56.1	43.9	<0.001
*Female*	50.4	49.6	
**Age**			
*18–20 years*	55.6	44.4	<0.001
*21–25 years*	49.0	51.0	
*26–30 years*	33.9	66.1	
**Socio-economic status**			
*Low*	41.8	58.2	<0.001
*High*	61.5	38.5	
**Living during term time**			
*Parents’ house*	52.2	47.8	<0.001
*Relatives’ house*	19.7	80.3	
*College residence*	49.8	50.2	
*Rented house/flat*	64.7	35.3	
*Other*	57.6	42.4	
**Academic Discipline**			
*Natural Sciences*	56.8	43.2	<0.001
*Social Sciences*	56.9	43.1	
*Humanities*	56.7	43.3	
*Applied Sciences: Medicine and Healthcare professions*	43.2	56.8	
*Applied Sciences: Engineering and technology*	53.0	47.0	
*Business and economics*	59.4	40.6	
*Professions (e.g., architecture)*	24.9	75.1	
*Formal Sciences: information technology, mathematics*	37.0	63.0	

**TABLE 2:** Health-related behavior among university students with and without depressive symptoms (*N* = 4228).

	Not clinically relevant (BDI-S <35) %	Clinically relevant (BDI-S ≥35) %	*p*-value
**Physical activity**			
*Low*	50.5	49.5	<0.001
*Moderate*	44.9	55.1	
*High*	67.6	32.4	
**Heavy episodic drinking**			
*Low-risk drinkers*	48.2	51.8	<0.001
*High-risk drinkers*	56.6	43.4	
**Suicide ideation**			
*Never*	71.6	28.4	<0.001
*Atleast once*	15.8	84.2	
**Self-rated health**			
*Good*	55.0	45.0	<0.001
*Fair/poor*	38.7	61.3	
**Smoking status**			
*Non-smoker*	43.7	56.3	<0.001
*Ever smoker*	55.2	44.8	
**Perceived Stress**			
*Low*	94.1	5.9	<0.001
*Moderate*	46.2	53.8	
*High*	12.0	88.0	
**Social Capital: Behavioral Dimension**			
*Low*	43.2	56.8	<0.001
*High*	66.1	33.9	
**Social Capital: Cognitive Dimension**			
*Low*	34.5	65.5	<0.001
*High*	54.1	45.9	

**FIGURE 1:**
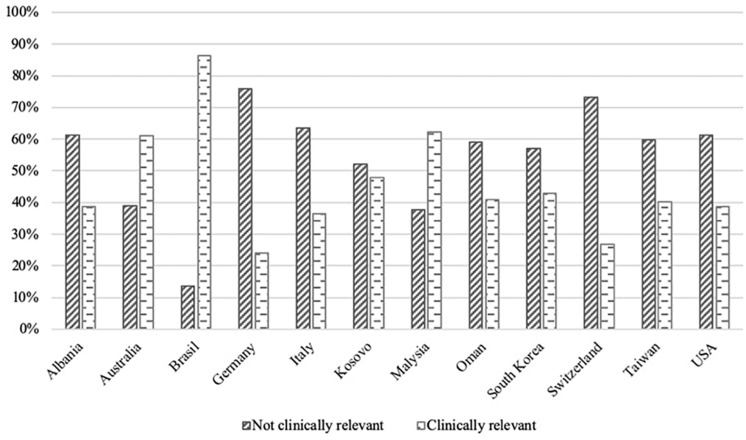
Prevalence of depressive symptoms by country.

The majority of the participants were female (64.7%). Forty-eight percent of students scored positive on clinically relevant depressive symptoms (BDI-S ≥ 35) (Table 1). Prevalence estimates of clinically relevant depressive symptoms ranged from a low of 24% in Germany to a high of 86% in Brazil (Figure 1).

A significantly higher proportion of students with clinically relevant depressive symptoms were from families with lower socioeconomic backgrounds (*p* < 0.001) (Table 1). Students with a lower stock of social capital had higher prevalence of depressive symptoms (*p* < 0.001) than students with higher levels social capital levels (Table 2).

#### Factors Associated With Depressive Symptoms

Results of the multilevel logistic models are shown in Table 3. After controlling for potential confounding factors (age, gender, and socioeconomic status), both low levels of cognitive (OR: 1.82, 95%CI: 1.44–2.29) and behavioral social capital (OR: 1.51, 95%CI: 1.29–1.76) were significantly associated with clinically relevant depressive symptoms (Model 1). The unadjusted and adjusted analysis yielded approximately the same magnitude of effects suggesting that age, sex, and SES may not be a major confounding factor to the association between social capital and depressive symptoms.

**TABLE 3:** Results for multilevel models, displaying adjusted odds-ratios (OR) and 95% confidence intervals (CI) for social capital and depressive symptoms.

	Model 1	Model 2	Model 3
	OR (95% CI)	OR (95% CI)	OR (95% CI)
**Social capital behavioral dimension**			
*High social capital (Ref)*	1.00	1.00	1.00
*Low social capital*	1.51 (1.29–1.76)	1.45 (1.21–1.74)	1.48 (1.23–1.77)
**Social capital cognitive dimension**			
*High social capital (Ref)*	1.00	1.00	1.00
*Low social capital*	1.82 (1.44–2.29)	1.67 (1.27–2.22)	1.65 (1.25–2.20)
**Gender**			
*Male (Ref)*	1.00	1.00	1.00
*Female*	1.36 (1.16–1.60)	1.09 (0.90–1.32)	1.10 (0.91–1.33)
**Age**	1.00 (1.00–1.00)	1.00 (1.00–1.00)	1.00 (1.00–1.00)
**Socioeconomic Status**			
*High (Ref)*	1.00	1.00	1.00
*Low*	1.45 (1.24–1.70)	1.32 (1.11–1.58)	1.33 (1.11–1.58)
**Self-Rated Health**			
*Good (Ref)*		1.00	1.00
*Poor/Fair*		2.50 (1.94–3.22)	2.49 (1.93–3.21)
**Perceived Stress**			
*Low Stress (Ref)*		1.00	1.00
*High Stress*		17.57 (11.33–27.26)	17.65 (11.38–27.39)
**Smoking Status**			
*Non-Smoker (Ref)*		1.00	1.00
*Ever Smoker*		1.06 (0.81–1.38)	1.06 (0.82–1.39)
**Heavy Episodic drinking**			
*Low-risk drinkers (Ref)*		1.00	1.00
*High-risk drinkers*		0.99 (0.75–1.13)	0.99 (0.80–1.24)
**Physical Activity**			
*Low (Ref)*		1.00	1.00
*Moderate*		0.92 (0.75–1.13)	0.92 (0.75–1.13)
*High*		0.62 (0.49–0.78)	0.62 (0.49–0.79)
**Country-Level Characteristics/Contextual Factors**			
**Level of Income**			
*High-income economy (Ref)*			1.00
*Lower to upper-middle income economy*			3.47 (1.43–8.42)
**ICC**	0.19 (0.09–0.36)	0.22 (0.11–0.41)	0.17 (0.08–0.33)

*Adjusted for age, sex and SES; *Ref* = *Reference group.**

The odds of reporting clinically relevant depressive symptoms were significantly higher among students with high perceived stress (OR: 17.57, 95%CI: 11.33–27.26) than students with lower perceived stress. A higher level of physical activity per week was inversely associated with depressive symptoms (OR: 0.62, 95%CI: 0.49–0.78).

When accounting for country-level characteristics, the analyses showed that students living in lower to upper-middle-income economies (e.g., Albania, Brazil, Kosovo, Malaysia) had higher odds (OR: 3.47, 95%CI: 1.43–8.42) of reporting clinically relevant depressive symptoms (Table 3, Model 3). In an additional analysis, aggregating responses of trust (proportion of students agreeing that people can be trusted), we found that individuals living in countries with low levels of trust had a significantly higher risk of depressiveness (OR: 2.87, 95%CI: 1.08–7.58) (Supplementary Table S4). Low trust countries included: Albania, Australia, Brazil, Italy, Kosovo and Malaysia.

Sensitivity tests revealed that neither excluding countries with very high prevalence estimates (Supplementary Table S2) nor using the BDI-S scores (Supplementary Table S3) as a continuous variable substantially change our findings. When excluding countries with very high rates of clinically relevant depressive symptoms, students with a lower stock of cognitive social capital (OR: 1.90, 95%CI: 1.47–2.46) and behavioral social capital (OR: 1.38, 95%CI: 1.16–1.63) were still significantly more likely to report clinically relevant depressive symptoms (Supplementary Table S2). When using the BDI-S scores as a continuous variable results still showed that lower levels of cognitive social capital (*ß* = 5.90, *p* < 0.001) and behavioral social capital (*ß* = 4.12; *p* < 0.001) were predictors for clinically relevant depressive symptoms, confirming the findings of the logistic regression analysis (Supplementary Table S3).

Without including any individual-level characteristics variable, 19% of the variance in individual depressive symptoms came from the country level (Table 3, Model 1). After adding individual-level characteristics, the variance increased to 22% (Table 3, Model 2).

## Discussion

This study investigated the prevalence of depressive symptoms and the importance of social capital on depressive symptoms in university students from twelve countries. The prevalence of depressive symptoms was high (48%), with considerably high rates among students from Brazil (86%). The overall prevalence of depressive symptoms in our study is consistent with the average rates reported in previous research (Ibrahim et al., 2013; Auerbach et al., 2018). Auerbach et al. (2018), for instance, found that the 12-month prevalence estimates of common mental disorders ranged from a low of 22% in Belgium to a high of about 48% in Australia.

Furthermore, in the present research, we expand current literature on the prevalence of depressive symptoms among university students and examined the association between social capital and depressive symptoms. Findings supported our primary hypothesis that students with a lower stock of social capital experience greater depressive symptoms.

As expected, at the individual-level social capital is significantly associated with clinically relevant depressive symptoms, also after accounting for age, gender and family socio-economic status. Meaning that students with low individual perceptions of social capital are at greater risk of clinically relevant depressive symptoms. These results are in line with prior studies that have shown significant associations between individual-level social capital and mental health and self-rated health (Borges et al., 2010; Cohen-Cline et al., 2018).

The results of the multilevel logistic regression analyses also put forward important macro-level aspects of social capital. Students living in lower to upper-middle income countries presented higher odds of presenting clinically relevant depressive symptoms. Moreover, countries, in which students had the lowest level of social capital also had the highest levels of depressive symptoms. An additional examination revealed that the tendency to report distrust was highest among students in Albania, Brazil and Malaysia, while those in Germany and Switzerland reported the lowest level distrust. One could speculate over the reasons for these international differences, but they are likely to be the product of quite particular political, historical and social and cultural factors. In particular, because these countries seem to be ones with high levels of corruption, ethnic conflict, political repression, instability and upheaval. Brazil, for example is characterized by its inherent socioeconomic inequalities with gaps among different social classes, general distrust in both people and in the government, economic recession, ideological polarization, loss of purchasing power, cuts in public investments (Vincens et al., 2018). All which have been found to be detrimental for mental health (van Deurzen, 2017). Debates on the welfare and labor reforms are on-going and underinvestment in public services has caused frustration and anger amongst Brazilians (The Fund for Peace, 2019). Similar contextual factors can be found in Albania, Kosovo and Malaysia. Kosovo, as developing country and Albania, as one of the poorest countries in Europe, are characterized by high rates of unemployment, poverty, social exclusion and gender disparities (World Bank, 2018). In Malaysia ethnic inequality and income seem to persist, although the Gini coefficient has dropped considerably over the past years. All factors represent main causes of mental health issues (Marmot, 2014).

Concerning the very high rates of depressive symptoms in Brazil, it is important to take the location of the University of Brasília into account. Brasilia is the federal capital of Brazil and the political hub of the country experiencing great economic and political difficulties (Vincens et al., 2018). Furthermore, the survey in Brazil was conducted after the election of President Bolsanaro, whose administration has made concerted attacks on universities, including cuts to financial aid for disadvantaged students. At the University of Brasília, yet, around 70% of students receive scholarships and/or depend on FIES. Thus, many students were faced with uncertainty regarding the future of their education. Previous studies have reported that financial difficulties can have a strong and independent effect on depression (Economou et al., 2013).

Several theoretical explanations may account for the findings regarding associations between depressiveness and social capital (Kawachi and Berkman, 2001; Fujiwara and Kawachi, 2008). The stress buffering model, for example, theorizes that social capital can provide opportunities for (psycho) social support which may act as a ‘buffering factor’ for stress (Kawachi and Berkman, 2001). The main effect model hypothesizes that living in a highly trusting environment can have a protective effect against mental illness (Kawachi and Berkman, 2001). Moreover, considering social capital at the macro-level, specifically, it is possible that countries with high levels of social capital have better health because they have better public services (Halpern, 2005). Researchers have put forward that there is a reciprocal relationship between state-level social capital and government performance (Halpern, 2005). Furthermore, higher social trust has been associated with lower rates of government corruption and better infrastructure (La Porta et al., 1997). A second possibility is that the effect might relate to shared mutual norms and values. It is possible that in high-trust countries people are nicer to one another, are more supportive and that life, in general, is less conflictual. The third possibility includes income inequality (Kawachi et al., 1997). Kawachi et al. (1997) noted a strong correlation between income inequality and both per capita group membership and lack of social trust (Kawachi et al., 1997). Less well-off individuals may be less likely to subscribe to social groups such as sports clubs. Therefore, it is possible, that increased income inequality reduces social capital which in turn results in poorer health in the relevant groups. The numbers of studies that directly investigate whether cross-national differences in health can be explained by cross-national variations in social capital is limited. The strongest evidence for macro-level social capital having an impact on health probably comes from Kawachi et al. (1997) and Helliwell (2004). Kawachi et al. (1997), discovered that United States states which had higher levels of social mistrust had higher levels of all age-adjusted total mortality and higher rates of fair/poor health (Kawachi et al., 1997). Helliwell (2004) found a close relationship between social capital and suicide (Helliwell, 2004).

### Implications

There are several implications that render from these results. First, targeting young people remains fundamental because poor health can seriously affect students’ education, an essential determinant of health. Higher educational attainment, in particular, has been associated with better social and economic development (e.g., higher income) and with an increase of one’s capacity for better decision-making regarding health (Marmot, 2017).

Given the high rates of depressive symptoms there are a few implications directed toward universities. Higher levels of social capital may have positive effects on students’ mental health. Therefore, universities should consider strengthening and implementing interventions focusing on enhancing social capital. This could involve, for example, promoting social and sports clubs to reduce social isolation or promoting students to refrain from time-out on phones, but time-in with conversations with friends and classmates. Furthermore, there is an urgent need for on-campus mental health counseling services. The concept of social capital should be considered as an add-on component in mental health interventions.

Last, given the macro-level findings, it is possible that students’ level of mental health reflects broader social and political problems in society. Therefore, the policy prescription would not only be to improve individuals’ social capital, but to turn the focus on wider social as well political contexts when analyzing students’ health.

### Strengths and Limitations

A major strength of the study is that it fills a gap in the current literature by offering new insight into students’ mental health and data on social capital. The international multicenter study design enabled us to make comparisons across countries and to contribute to the discussion of the macro-level aspects of social capital. However, the study has limitations that must be considered. First, the cross-sectional data precludes any inferences of causality or directionality of the effects of social capital on health. A lower stock of social capital may lead to reduced levels of health, but poor health could also generate more moderate levels of social capital. Therefore, longitudinal studies investigating social capital and depressive symptoms are needed. Second, data were obtained through self-reported questionnaires and self-reporting and recall bias for both mental health and social capital cannot be excluded. Over- or underreporting which may have inflated or deflated the associations between social capital and depressiveness is possible. Third, only one to two universities per country participated in the study. Therefore, the sample may not be completely representative of the entire student population in each country. Furthermore, this did not allow us to include an additional level (i.e., university level) in the multilevel analysis. This meant that we could not study campus-level social capital as a predictor of mental health status, and that some portion of the country-level variance in the outcome was actually attributable to campus-level variance.

Nonetheless, at this point of time this sample is larger than in most other epidemiological studies investigating students’ health (Barker et al., 2018; Ngin et al., 2018). Nevertheless, it would be beneficial to replicate the study in more universities in each country. Last, although we adjusted for a large number of factors, some variables such loss of a close family member or family history of depression, that could contribute to depressiveness, were not assessed.

## Conclusion

This study identified that lower levels of social capital, at both the micro-level and macro-level, are associated with depressive symptoms among college students. Students’ poor mental health might reflect broader social and political problems in society. While we tend to think of college students as being a protected and privileged group in society, the findings suggest that they can also be “canaries in the coalmine.” Continued effort on the identification of specific factors that improve or worsen students’ mental health is needed in order to better understand the onset and course of illness and to develop effective prevention and intervention strategies.

## Data Availability Statement

The dataset for this study is available on request to the corresponding author.

## Ethics Statement

The present study received ethical approval by Institutional Review Boards and Ethics Committees at all participating institutions.

## Author Contributions

IB conceived the idea of the study. IB, AV, JW, FF, SK, EB, GL, and IK designed the study. JW and FF collected the data at Bielefeld University (Germany). KS and AA-S collected the data at the University of South Australia (Australia). AV and LM collected the data at the University of Brasília (Brazil). EB collected the data at the University of Zurich (Switzerland), the University of Tirana (Albania), and University for Business and Technology (Kosovo). BL collected the data at the Baylor University (United States). AA-S collected the data at the Sohar University (Oman). P-HL collected the data at the National Taiwan Normal University (Taiwan). SK collected the data at the University of Malaya (Malaysia). IB collected the data at the Harvard T.H. Chan School of Public Health (United States) and the Sapienza University of Rome (Italy), conducted the statistical analyses with the help of GL and IK, and wrote the first draft of the manuscript with the support from all authors. All authors significantly participated in interpreting the results, revising the manuscript, and approved its final version.

## Conflict of Interest

The authors declare that the research was conducted in the absence of any commercial or financial relationships that could be construed as a potential conflict of interest.
