# Supplementary material for: Associations Between Social Capital and Depressive Symptoms Among College Students in 12 Countries: Results of a Cross-National Study
Source: Front Psychol. 2020 Apr 29;11:644. doi: 10.3389/fpsyg.2020.00644 (PMC7201069; doi:10.3389/fpsyg.2020.00644)
Supplement: Supplementary file 1 [file Table_1.DOCX]

**Supplemental material**

**Sample size calculation**

The sample size was calculated with a sensitivity of 95%, a margin of error of no more than ±5% and using the estimated prevalence of depressive symptoms (mild/moderate) in each country (i.e. each university). The following estimated prevalence rates of depressive symptoms (mild/moderate) were used for the calculations:

- 19.9% in Albania (Pilika & Simaku, 2017),
- 26.1% in Australia (Schofield et al., 2016),
- 14.0% in Brazil (Silva et al., 2014),
- 9.9% in Germany (Busch et al., 2013),
- 6.7% in Italy (Binkin et al., 2010),
- 25% in Kosovo (Džubur et al., 2018)
- 21.0% in Malaysia (Yeoh et al., 2017),
- 27.7% in Oman (Al-Busaidi et al., 2011),
- 21.4% in South Korea (Seo & Je, 2018),
- 18.9% in Switzerland (Baer et al., 2013),
- 32.6% in Taiwan (Chen et al., 2015), and
- 7.6% in the USA (Pratt & Brody, 2014).

Furthermore, the number of students enrolled at each university is considered (see below: Number of students enrolled at each institute). Based on the calculations, the following numbers of student should be sampled in each country:

- n= 243 students from Albania,
- n= 290 students from Australia,
- n= 183 students from Brazil,
- n= 137 students from Germany,
- n= 96 students from Italy,
- n= 283 students from Kosovo,
- n= 252 students from Malaysia,
- n= 289 students from Oman,
- n= 256 students from South Korea,
- n= 234 students from Switzerland,
- n = 331 students from Taiwan, and
- n= 216 students from the USA (n=108 Harvard University, n=108 Baylor University).

The following formula was used to calculate the sample size:

$$n= \frac{n}{1+(\frac{z^{2}x p(1-p)}{e^{2}N}}$$

Assuming a possible incomplete data rate of 20%, the final sample for each university/country is:

- n1= 303 students from Albania,
- n1= 362 students from Australia,
- n1= 228 students from Brazil,
- n1= 171 students from Germany,
- n1= 120 students from Italy,
- n1 = 353 students from Kosovo,
- n1= 315 students from Malaysia,
- n1= 361 students from Oman,
- n1= 320 students from South Korea,
- n1= 292 students from Switzerland,
- n1= 413 students from Taiwan, and
- n1= 270 from the USA (n=216 Harvard University, n=216 Baylor University) should be sampled.

The overall sample size for this study is therefore n= 3,508 students. The following formula was used to calculate the sample size considering missing data:

$$n1= \frac{n}{\left( 1-\left( \frac{z}{100} \right) \right)}$$

**Number of students enrolled at each institute**

**University of Tirana, Albania**

The University of Tirana is a public and the largest university in Albania. An estimated number of over 14.000 students attend this university.

**University of South Australia, Australia**

The University of South Australia is a public research university in the Australian state of South Australia. It is the largest university in South Australia with almost 32.000 students.

**University Center of the Federal District, Brazil**

The University Center of the Federal District (UDF) in Brasilia was created in 1967 and is the first private graduate education institution in the Brazilian capital. There are almost 16.000 students, 300 teachers, and 235 employees, distributed in an undergraduate and postgraduate course.

**Bielefeld University, Germany**

The Bielefeld University is located in North Rhine–Westphalia and is one of the country's newer universities and has over 24.000 students.

**Sapienza University of Rome, Italy**

The Sapienza University of Rome is a collegiate research university with 11 faculties and is the largest European university by enrolments (n= students **110.000 in 2015/16)**.

**University of Malaya, Malaysia**

The University of Malaya is a public research university located in Kuala Lumpur, Malaysia. It is the oldest and most esteemed University in Malaysia. Around 17.000 students are enrolled.

**Sohar University, Oman**

Sohar University was established in 2001 as the first private university in Oman. Around 5.000 students are enrolled.

**Chung-Ang University, South Korea**

Chung-Ang University is a Korean private university based in Seoul, South Korea with around 21.8000 students.

**University of Zurich, Switzerland**

The University of Zurich in the city of Zurich is located, in the canton of Zurich. With over 26.0000 students, it is the largest university in Switzerland.

**National Taiwan University, Taiwan**

The National Taiwan University is a national university in Taipei City. It is considered the most prestigious university in Taiwan and one of the top-ranked universities in the world, and nearly 33.000 students are enrolled.

**Harvard University, United States of America**

The Harvard University is a private Ivy League research university in Cambridge, Massachusetts, with about 21.000 students enrolled.

**Baylor University, United States of America**

Baylor University is a private Christian university in Waco, Texas with about 16.787 students enrolled.

**Table S1. Sample size, mean age and for each country**

| Country | N (%) | Mean age +/- SD |
| --- | --- | --- |
| Albania | 258 (6.1) | 18.9 (1.5) |
| Australia | 397 (9.4) | 19.57 (2.5) |
| Brazil | 549 (13.0) | 23.05 (3.5) |
| Germany | 708 (16.7) | 19.92 (2.1) |
| Italy | 402 (9.5) | 19.39 (1.4) |
| Kosovo | 142 (3.4) | 19.10 (1.6) |
| Malaysia | 444 (10.5) | 20.39 (1.5) |
| Oman | 278 (6.6) | 19.94 (2.1) |
| South Korea | 319 (7.5%) | 22.87 (1.7) |
| Switzerland | 251 (5.9) | 20.69 (7.9) |
| Taiwan | 214 (5.1) | 19.26 (1.4) |
| USA campus 1  USA campus 2 | 200 (4.7)  66 (1.6) | 26.2 (2.2)  18.17 (0.3) |

**Table S2.** Sensitivity Analysis excluding countries with very high rates of depressive symptoms

|  | Model 1 | | Model 2 | | Model 3 | | |
| --- | --- | --- | --- | --- | --- | --- | --- |
|  | **OR (95% CI)** | | **OR (95% CI)** | | **OR (95% CI)** | | |
| Social capital Behavioral Dimension | | | | | | | |
| *High social capital (Ref)* | 1.00 |  | 1.00 |  | 1.00 |  | |
| *Low social capital* | 1.38 (1.16 **-** 1.63) | | 1.32 (1.08 - 1.61) | | 1.33 (1.09 - 1.62) | | |
| Social capital Cognitive Dimension | | | | | | | |
| *High social capital (Ref)* | 1.00 |  | 1.00 | | 1.00 |  | |
| *Low social capital* | 1.90 (1.47 **-** 2.46) | | 1.87 (1.36 - 2.58) | | 1.87 (1.36 - 2.58) | | |
| Gender | | | | | | | |
| *Male (Ref)* | 1.00 |  | 1.00 |  |  | | |
| *Female* | 1.42 (1.19 - 1.71) | | 1.34 (0.92 - 1.42) | | 1.14 (0.91 - 1.42) | | |
| *Other than male or female* | 1.72 (0.86 - 3.43) | | 1.60 (0.75 - 3.40) | | 1.59 (0.77 - 3.28) | | |
| Age | 1.00 (0.98 - 1.02) | | 1.00 (0.98 - 1.04) | | 1.00 (0.98 - 1.04) | | |
| Socioeconomic status | | | | | | | |
| *High (Ref)* | 1.00 |  | 1.00 |  | 1.00 |  | |
| *Low* | 1.49 (1.26 - 1.76) | | 1.36 (1.11 - 1.65) | | 1.34 (1.10 - 1.64) | | |
| Self-rated health | | | | | | | |
| *Good (Ref)* |  | | 1.00 |  | 1.00 |  | |
| *Poor/fair* |  | | 2.51 (1.91 - 3.29) | | 2.52 (1.92 - 3.30) | | |
| Perceived stress | | | | | | | |
| *Low stress (Ref)* |  | | 1.00 |  | 1.00 |  | |
| *High stress* |  | | 18.21 (11.51 - 28.81) | | 18.28 (11.55 - 28.92) | | |
| Smoking status | | | | | | | |
| *Non-smoker (Ref)* |  | | 1.00 |  | 1.00 |  | |
| *Ever smoker* |  | | 1.21 (0.92 - 1.61) | | 1.24 (0.94 - 1.64) | | |
| Alcohol consumption | | | | | | | |
| *Non-hazardous (Ref)* |  | | 1.00 |  | 1.00 |  | |
| *Hazardous* |  | | 1.09 (0.86 -1.38) | | 1.10 (0.87 - 1.40) | | |
| Physical activity | | | | | | | |
| *Low (Ref)* |  | | 1.00 |  | 1.00 |  | |
| *Moderate* |  | | 0.89 (0.71 - 1.13) | | 0.90 (0.71 - 1.14) | | |
| *High* |  | | 0.58 (0.43 - 0.74) | | 0.56 (0.43 - 0.74) | | |
| Country-level characteristics | | | | | | | |
| *High income economies (Ref)* |  | |  | | 1.00 | |  |
| *Lower-to upper-middle-income economies* |  | |  | | 2.10 (0.83 - 5.32) | | |
| ICC | 0.05 (0.02 - 0.12) | | 0.06 (0.02 - 0.16) | | 0.04 (0.01 - 0.13) | | |

**Table S3.** Linear regression analysis for variables predicting depressive symptoms*

|  | **Model 1** | | | | **Model 2** | | | **Model 3** | | |
| --- | --- | --- | --- | --- | --- | --- | --- | --- | --- | --- |
| **Variable** | *β* | *SE* | | *p-value* | *β* | *SE* | *p-value* | *β* | *SE* | *p-value* |
| **Low Social capital Cognitive Dimension** | 5.90 | 0.97 | | <0.001 | 4.36 | 0.98 | <0.001 | 4.31 | 0.98 | <0.001 |
| **Low Social capital Behavioral Dimension** | 4.12 | 0.68 | | <0.001 | 3.30 | 0.67 | <0.001 | 3.28 | 0.67 | <0.001 |
| **High perceived stress** |  |  | |  | 23.22 | 1.13 | <0.001 | 23.29 | 1.13 | <0.001 |
| **Fair/poor Self-rated health** |  |  | |  | 6.89 | 0.86 | <0.001 | 6.86 | 0.86 | <0.001 |
| **Ever smoker** |  |  | |  | 1.76 | 0.87 | 0.044 | 1.84 | 0.86 | 0.033 |
| **Hazardous alcohol consumption** |  |  | |  | -1.54 | 0.75 | 0.041 | -1.49 | 0.75 | 0.046 |
| **Physical activity** |  | | | | | | | | | |
| *Moderate* |  | |  |  | -1.13 | 0.78 | 0.146 | -1.10 | 0.78 | 0.159 |
| *High* |  | |  |  | -2.68 | 0.88 | 0.002 | -2.63 | 0.88 | 0.003 |
| **Country-level** |  | | | | | | | | | |
| *Upper-middle-income economies* |  | |  |  |  |  |  | 4.72 | 2.31 | 0.041 |
| *Lower-middle-income economies* |  | |  |  |  |  |  | 4.04 | 4.02 | 0.315 |

Note: Adjusted for age, sex and SES

**Table S4. Results for supplemental multilevel models, displaying adjusted odds-ratios* (OR) (95% CI) for social capital and depressive symptoms**

|  | **Model 1** | | **Model 2** | | **Model 3** | |
| --- | --- | --- | --- | --- | --- | --- |
|  | **OR (95% CI)** | | **OR (95% CI)** | | **OR (95% CI)** | |
| **Social capital behavioral dimension** | | | | | | |
| *High social capital (Ref)* | 1.00 |  | 1.00 |  | 1.00 |  |
| *Low social capital* | **1.51 (1.29 - 1.76)** | | **1.45 (1.21 - 1.74)** | | **1.44 (1.20 - 1.72)** | |
| **Social capital cognitive dimension** | | | | | | |
| *High social capital (Ref)* | 1.00 |  | 1.00 | | 1.00 |  |
| *Low social capital* | **1.82 (1.44 - 2.29)** | | **1.67 (1.27 - 2.22)** | | **1.67 (1.26 - 2.20)** | |
| **Gender** | | | | | | |
| *Male (Ref)* | 1.00 |  | 1.00 |  | 1.00 |  |
| *Female* | **1.36 (1.16 - 1.60)** | | 1.09 (0.90 - 1.32) | | 1.13 (0.94 - 1.36) | |
| **Age** | 1.00 (1.00 - 1.00) | | 1.00 (1.00 - 1.00) | | 0.98 (0.94 - 1.02) | |
| **Socioeconomic Status** | | | | | | |
| *High (Ref)* | 1.00 |  | 1.00 |  | 1.00 |  |
| *Low* | 1.45 (1.24 - 1.70) | | 1.32 (1.11 - 1.58) | | 1.33 (1.11 - 1.58) | |
| **Self-Rated Health** | | | | | | |
| *Good (Ref)* |  | | 1.00 |  | 1.00 |  |
| *Poor/Fair* |  | | **2.50 (1.94 - 3.22)** | | **2.54 (1.97- 3.21)** | |
| **Perceived Stress** | | | | | | |
| *Low Stress (Ref)* |  | | 1.00 |  | 1.00 |  |
| *High Stress* |  | | **17.57 (11.33 - 27.26)** | | **17.16 (11.15 - 26.40)** | |
| **Smoking Status** | | | | | | |
| *Non-Smoker (Ref)* |  | | 1.00 |  | 1.00 |  |
| *Ever Smoker* |  | | 1.06 (0.81 - 1.38) | | 1.06 (0.82 - 1.38) | |
| **Alcohol Consumption** | | | | | | |
| *Non-Hazardous (Ref)* |  | | 1.00 |  | 1.00 |  |
| *Hazardous* |  | | 0.99 (0.75 - 1.13) | | 0.83 (0.83 - 1.28) | |
| **Physical Activity** |  | |  | |  | |
| *Low (Ref)* |  | | 1.00 |  | 1.00 |  |
| *Moderate* |  | | 0.92 (0.75 - 1.13) | | 0.90 (0.73 - 1.28) | |
| *High* |  | | **0.62 (0.49 - 0.78)** | | **0.63 (0.50 - 0.80)** | |
| **Country-Level Characteristics/Contextual Factors** | | | | | | |
| **Level of Trust** |  | |  | |  | |
| *High trust (Ref)* |  | |  | | 1.00 |  |
| *Low trust* |  | |  | | **2.87 (1.08 - 7.58)** | |
| **ICC** | 0.19 (0.09 - 0.36) | | 0.22 (0.11 - 0.41) | | 0.16 (0.07 - 0.32) | |
